# Prognostic sub-classification of intermediate-stage hepatocellular carcinoma: a multicenter cohort study with propensity score analysis

**DOI:** 10.1007/s12032-016-0827-8

**Published:** 2016-09-06

**Authors:** Ramya Ramaswami, David J. Pinato, Keiichi Kubota, Mitsuru Ishizuka, Tadaaki Arizumi, Masatoshi Kudo, Jeong Won Jang, Young Woon Kim, Mario Pirisi, Elias Allara, Rohini Sharma

**Affiliations:** 1 Department of Surgery and Cancer, Imperial College London Hammersmith Hospital, Du Cane Road, London, W12 0HS UK; 2 Department of Medical Oncology, The Royal Marsden NHS Foundation Trust, Downs Road, Sutton, SM2 5PT UK; 3 Department of Surgery, Dokkyo Medical University, Dokkyo, Japan; 4 Department of Gastroenterology and Hepatology, Kinki University School of Medicine, Osaka-Sayama, Osaka Japan; 5 Department of Internal Medicine, The Catholic University of Korea Incheon St. Mary’s Hospital, Seoul, Republic of Korea; 6 Department of Translational Medicine, Università degli Studi del Piemonte Orientale “A. Avogadro”, Via Solaroli 17, 28100 Novara, Italy; 7 Interdisciplinary Research Center of Autoimmune Diseases, Università degli Studi del Piemonte Orientale “A. Avogadro”, Via Solaroli 17, 28100 Novara, Italy

**Keywords:** Hepatocellular cancer, Transarterial chemoembolization, Liver resection, Prognosis, Multicenter

## Abstract

There is significant heterogeneity in the clinicopathological characteristics of intermediate hepatocellular carcinoma (IHCC). This also translates to treatment as transarterial chemoembolization (TACE) is used as first-line therapy for patients with IHCC; however, in Asia liver resection (LR) is preferred. Prognostic tools are required to help guide clinicians in deciding treatment options. This study evaluates the prognostic impact of the Intermediate Stage Score (ISS) on overall survival (OS) in a large, multicenter cohort study of patients with IHCC treated with TACE or surgery LR. Consecutive patients from centers in Japan, Korea, Italy and the United Kingdom who underwent TACE or LR between 2001 and 2015 were enrolled. Propensity score (PS) adjustment was used to remove residual confounding and applied to LR (*n* = 162) and TACE (*n* = 449) to determine the prognostic significance of ISS. Among 611 patients, 75 % were men and 25 % women, with a mean age of 70 years. ISS is a valid prognostic tool in the BCLC-B population with a median OS ISS 1–51, 2–38.3, 3–24.3, 4–15.6, 5–16 months (*p* < 0.0001). ISS was analyzed within each treatment modality, and this was a valid prognostic score among those treated with TACE and LR (*p* < 0.001 vs. *p* = 0.008). In the PS-adjusted model, ISS retained its prognostic utility in TACE and LR groups (*p* < 0.001 vs. *p* = 0.007). ISS optimizes prognostic prediction in IHCC, reducing clinical heterogeneity, and is a useful tool for patients treated for TACE or LR.

## Introduction

Hepatocellular carcinoma (HCC) is one of the leading causes of cancer-related death in patients with liver cirrhosis, with more than new 700,000 cases diagnosed yearly worldwide [1, 2]. Over the past few decades, it has become clear that the natural history of HCC strongly depends on anatomical stage, underlying liver function and overall patients’ physical status: this has led to the development of several prognostic algorithms with intent to optimize treatment [3–7].

The Barcelona Clinic Liver Cancer (BCLC) stage includes prognostic variables such as tumor stage, performance status, and Child–Turcotte–Pugh (CTP) class [8]. Prospective validation of the BCLC staging system has demonstrated reliable prognostic subdivision of HCC [9, 10]. Due to its association with treatment allocation, the BCLC algorithm has received formal endorsement by organizations such the European Association for the Study of the Liver (EASL) and the American Association for the Study of Liver Diseases (AASLD) [11–13]. However, there is marked heterogeneity in the reported 3-year survival in BCLC-B stage disease of 10–40 %. Therefore, formulating appropriate treatment strategies for the individual patient is difficult within this nebulous BCLC-B staging system.

According to the BCLC staging system, transarterial chemoembolization (TACE) is recommended as first-line treatment for patients with IHCC or BCLC-B. Two randomized controlled trials have shown an approximate 50 % reduction in mortality in patients treated with TACE compared to controls [14, 15]. A significant OS benefit from TACE has been further consolidated by two separate meta-analyses [16], which however re-defined the magnitude of benefit of TACE due to patient and procedural heterogeneity, resulting in some of the pooled studies not meeting their primary survival endpoints [17].

Issues such as the relative efficacy of TACE and the risk of adverse events among this group of patients results in the use of sorafenib, trial therapies or best supportive care [18, 19]. Alternatively, clinicians who do not adhere to BCLC guidelines offer other treatments such as resection or transarterial radioembolization (TARE) if IHCC patients meet local criteria [20, 21]. Therefore, despite the presence of consensus guidelines, there is variation in treatment in patients with BCLC-B disease. There is an urgent need for improved prognostication and subsequent stratification of management for patients with IHCC.

Bolondi et al. [22] created a prognostic score to further subdivide patients with IHCC in an effort to improve treatment allocation among this complex group. The Intermediate Stage Score (ISS) consists of five stages and includes CTP classification, ECOG performance status, portal vein thrombus and specific size criteria (Table 1). On the basis of the score, the authors recommended that patients can be offered first-line options such as TACE while patients with advanced stage (Quasi-C) should receive sorafenib [22]. There have been mixed outcomes in demonstrating the efficacy of this score. Two studies have demonstrated an association between ISS and OS among patients treated with bland transarterial embolization (TAE) and TACE (*N* = 580, 466) [23, 24]. However, in a separate European study, the score did not achieve prognostic significance (*N* = 254) [25]. Our intent was to validate the prognostic ability of the ISS in patients with intermediate-stage HCC (BCLC-B) by using propensity score analysis in diverse Eastern and Western populations treated with either surgical resection (LR) or TACE.

**Table 1:** BCLC-B sub-classification by Bolondi et al

BCLC sub-stage (ISS)	B1 (1)	B2 (2)	B3 (3)	B4 (4)	Quasi-C (5)
Child–Pugh score	5–6–7	5–6	7	8–9	5–6
Beyond milan and within Ut-7	In	Out	Out	Any	Any
ECOG PS	0	0	0	0–1	0
Portal vein thrombosis	No	No	No	No	Yes
1st line treatment	TACE	TACE or TARE		Best Supportive Care	Sorafenib
Alternative	LT TACE + Ablation	Sorafenib	Research trials TACE Sorafenib	LT	TACE or TARE

Proposed sub-classification and management recommendations for intermediate hepatocellular carcinoma as detailed by Bolondi et al. [15]

*BCLC* barcelona liver clinic, *ECOG* Eastern Cooperative Oncology Group, *PS* performance status, *LT* liver transplantation, *TACE* transarterial chemoembolization, *TARE* transarterial radioembolization

## Materials and methods

### Patient population

All centers in this study were involved in prospective collection of data from patients with a diagnosis of HCC made according to radiological or histological criteria, between 2001 and 2015. Patients were recruited from Hammersmith Hospital, London, St Mary’s Hospital, Seoul, University of Novara, and, Dokkyo Medical University, Dokkyo and Kinki University, Osaka). Informed consent was obtained from all patients recruited in this study in accordance with the Declaration of Helsinki and Good Clinical Practice (GCP) guidelines. Ethical approval for this study was obtained from the East London Research Ethics Committee.

Clinical variables were retrieved include patient demographics, complete blood count, albumin, aspartate and alanine aminotransferases (AST, ALT), alkaline phosphatase (ALP) alpha-fetoprotein (AFP), the international normalized ratio (INR) value and underlying etiology of liver disease was also identified. Patients with IHCC (BCLC-B) were categorized into five groups as per the criteria described by Bolondi et al. [22] (Table 1). Liver functional reserve was estimated using the CTP classification.

Tumor staging was described as the number of focal hepatic lesions and maximum diameter detected during contrast enhancement phase on computerized tomography. The Milan criteria and up-to-seven criteria (Up-to-7) were used to categorize size for calculating the ISS. The Milan criteria is defined as a single lesion <5 cm, up to three lesions <3 cm, the absence of gross vascular invasion or nodal or distant metastases [26]. Within the Up-to-7 criteria, seven is the sum of the size (centimeters) and the number of tumors for any given HCC [27].

### Statistical analysis

Continuous variables were presented as a median and range, and associations were tested using Mann–Whitney *U* or Student’s *t* test as appropriate. Categorical variables with absolute or relative frequencies were tabulated and or Fisher’s exact test, where appropriate. The OS rates for various ISS levels in all patients were analyzed using Kaplan–Meier method, and log-rank test was used to compare survival time. Univariate analyses of prognostic variables were completed with the Cox proportional hazards model. All statistical analyses were completed using two-sided test, and statistical significance was achieved where *p* < 0.05.

The date of HCC diagnosis till the date of death, loss to follow-up or study censoring (1st January 2016) was used to calculate overall survival. All patients were monitored with routine follow-up till the dates of death, loss to follow-up or study censoring.

Propensity score adjustment (PS) is a statistical method to reduce the effect of residual confounding in two groups [28]. In this study, PS was used to reduce the effect of residual confounding in the cohort by adjusting for confounding variables that are not accounted for within ISS classification, such as age, gender, hepatitis status and INR that impact treatment options. Cox regression analysis was used to determine the effect of ISS adjusted for PS quartiles in TACE and LR treatment groups. An interaction test was performed to determine the statistical significance of ISS in TACE and LR groups. Statistical analyses were performed using R version 3.1.2 (ww.r-project.org) and SAS 9.4 (SAS Institute Inc. Cary, North Carolina).

## Results

### Patient characteristics

Our study population consisted of 611 BCLC-B patients diagnosed with HCC across five centers (Table 2). The majority of patients underwent TACE (73.4 %) as first anticancer treatment, while 27.6 % were offered liver resection. Patients undergoing liver resection were younger (*p* < 0.001), while a higher proportion of patients undergoing TACE were Hepatitis B positive (*p* = 0.01). Five patients treated with TACE had portal vein thrombosis (PVT) and were classified as ‘Quasi C’ or ISS 5. There was a significant difference in the CTP classification between LR and TACE, with a higher proportion of patients with CTP > A6 receiving TACE (*p* < 0.01). The median OS (OS) of the overall population was 37 months (95 % confidence interval (CI) 33.0–39.3 months). The 1- and 3-year survival rates were 84.1, and 21.9 %, respectively. There was no significant difference between the median OS between TACE and LR subgroups (34.8 vs. 40 months, *p* = 0.09).

**Table 2:** Patient demographic at initial HCC diagnosis

Baseline characteristic	All patients (%), median, range *N* = 611	TACE intervention (%), median, range *N* = 449	LR intervention (%), median, range *N* = 162	*p* value
Age, years	70 (28–89)	72 (33–89)	68 (28–84)	<0.0001
Gender				0.39
Male	460 (75.3)	334 (74.4)	126 (77.8)	
Female	151 (24.7)	115 (25.6)	36 (22.2)	
Aetiology				
Hepatitis B infection	102 (16.7)	64 (14.3)	38 (23.4)	0.01
Hepatitis C infection	369 (60.4)	268 (59.7)	101 (62.3)	0.36
Alcohol related	97 (15.9)	97 (21.6)	–	–
Child–Turcotte–Pugh class				0.0003
A5	274 (44.8)	221 (49.2)	53 (32.7)	
A6	201 (32.9)	128 (28.5)	73 (45.0)	
B7	101 (16.5)	69 (15.4)	32 (19.8)	
B8	27 (4.4)	23 (5.1)	4 (2.5)	
B9	7 (1.2)	7 (1.5)	–	
Maximum tumor diameter				<0.0001
<7 cm	509 (83.3)	403 (89.8)	106 (65.4)	
≥7 cm	102 (16.7)	46 (10.2)	56 (34.6)	
Portal vein thrombus				–
Present	5 (1.1)	5 (1.1)	–	
Absent	444 (98.9)	444 (98.9)	–	
AFP, ng/mL	33 (1– > 1000)	32 (1– > 1000)	43.5 (1– > 1000)	0.44
Platelet count, ×10^9^/L	128 (26–470)	123 (26–453)	146 (44–470)	0.0008
ISS				<0.0001
1	104 (17.0)	42 (9.4)	62 (38.3)	
2	384 (62.8)	309 (68.8)	75 (46.3)	
3	84 (13.8)	63 (14.0)	21 (13.0)	
4	34 (5.6)	30 (6.7)	4 (2.5)	
5	5 (0.8)	5 (1.1)	–	
Median OS in months (95 % CI)	37 (33, 39.3)	34.8 (29.6, 38.9)	40 (34,47)	0.09

*AFP* alpha-fetoprotein, *INR* international normalized ratio, *BScore* scoring system for intermediate HCC, *OS* overall survival, *TACE* transarterial chemoembolization, *LR* liver resection

### ISS characteristics and OS

**Fig. 1:**
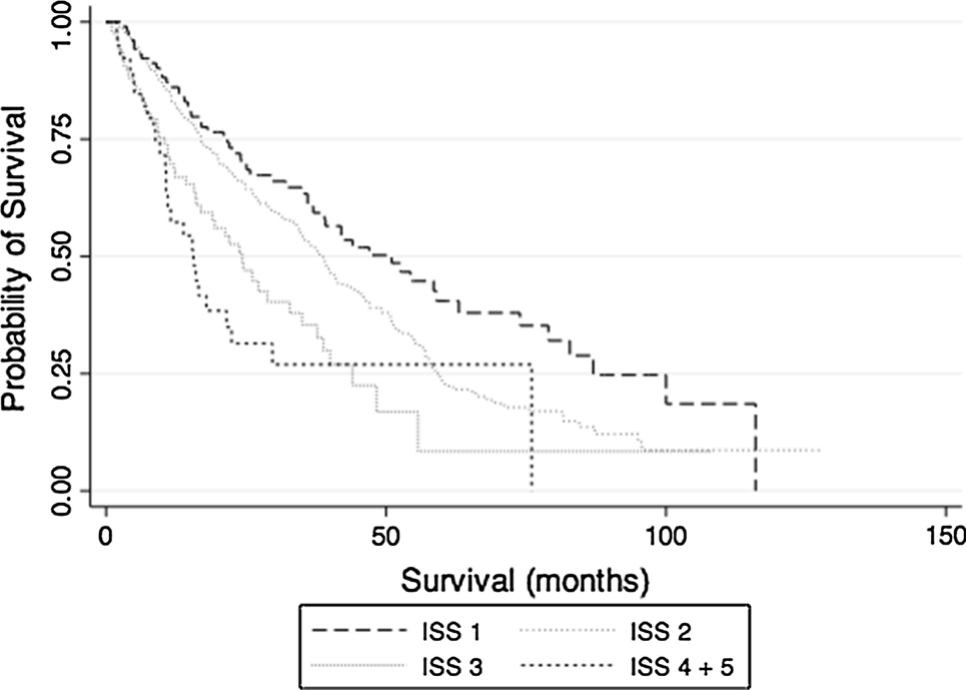
Cumulative mortality stratified by intermediate stage score (ISS) for all patients with intermediate hepatocellular carcinoma

In univariate analyses of the cohort, male gender, positive hepatitis B status and INR were variables that were significant for increased mortality and were not within the ISS prognostic score (Table 3). There was a difference in the ISS categories between TACE and LR groups, with a higher proportion of patients with ISS 2 or greater treated with TACE and those with an ISS of 2 or less treated with LR (*p* < 0.0001). There were no significant differences in baseline characteristics between ISS groups (Table 4). Due to the small number of patients with ISS 4 and 5, these were analyzed together to improve statistical validity. Significant differences in OS were observed between the different ISS groups ranging from 51 (ISS 1) to 16 months (ISS 4 and 5; *p* < 0.001), (Table 3; Fig. 1).

**Table 3:** Univariate analysis of factors that predict overall survival in patients with intermediate hepatocellular carcinoma (IHCC) treated with TACE or LR

Baseline characteristic	Hazard ratio (HR)	95 % confidence interval (CI)	*p* value
Age, years	1.01	0.99–1.01	0.32
Gender (F vs. M)	1.40	1.11–1.77	0.005
Aetiology			
Hepatitis B infection	0.69	0.51–0.93	0.01
Hepatitis C infection	1.23	0.99–1.53	0.06
Child–Turcotte–Pugh class			
A5			
A6	1.24	0.98–1.57	0.08
B7	1.62	1.20–2.19	0.002
B8	2.56	1.58–4.13	0.00
B9	3.22	1.19–8.72	0.02
Maximum tumor diameter (<7 vs. ≥7 cm)	1.11	0.86–1.43	0.42
Portal Vein Thrombus	1.51	0.48–4.71	0.48
AFP, ng/mL	1.00	0.99–1.00	0.07
Platelet Count, ×10^9^/L	0.99	0.996–0.999	0.03
ISS			
1	–	–	
2	1.39	1.03–1.87	0.03
3	2.29	1.55–3.39	0.00
4	3.19	1.95–5.23	0.00
5	2.27	0.71–7.29	0.17

**Table 4:** Sub-classification of BCLC-B with intermediate stage score (ISS) and corresponding characteristics

Factors	Total	ISS 1 (*n* = 104)	ISS 2 (*n* = 384)	ISS 3 (*n* = 84)	ISS 4 (*n* = 34)	ISS 5 (*n* = 5)	*p* value
Age, median years	70	67.4	69.7	68.9	66.1	65.8	0.07
Gender							0.088
Male	460 (75.3)	77 (74.0)	294 (76.6)	61 (72.6)	24 (70.6)	4 (80.0)	
Female	151 (24.7)	27 (25.9)	90 (23.4)	23 (27.3)	10 (29.4)	1 (20.0)	
Aetiology							
Hepatitis B infection	102 (16.7)	26 (25.0)	61 (15.9)	10 (11.9)	5 (14.7)	–	0.33
Hepatitis C infection	369 (60.4)	60 (57.7)	242 (63.0)	43 (51.2)	20 (58.8)	4 (80.0)	0.54
Alcohol related	97 (15.9)	11 (10.6)	57 (14.8)	19 (22.6)	9 (26.5)	1 (20.0)	0.34
Alpha-fetoprotein	33 (1– > 1000)	2279.4	5903.6	3923.2	1262.9	4525.5	0.96
Child–Turcotte–Pugh class							<0.0001
A5	274 (44.8)	53 (50.9)	219 (57.0)	–	–	2 (40.0)	
A6	201 (32.9)	35 (33.7)	164 (42.7)	–	–	1 (20.0)	
B7	101 (16.5)	16 (15.4)	–	84 (100)	–	2 (40.0)	
B8	27 (4.4)	–	–	–	27 (100)	–	
B9	7 (1.2)	–	–	–	7 (100)	–	
Maximum tumor diameter							0.0004
<7 cm	509 (83.3)	104 (100)	309 (80.5)	65 (77.4)	28 (82.4)	3 (60.0)	
≥7 cm	102 (16.7)	–	75 (19.5)	19 (22.6)	6 (17.6)	1 (20.0)	
Median overall survival in months	37	51	38.3	24.3	15.6	16	<0.0001

### ISS retains prognostic utility in propensity score adjustment analysis

When considering the prognostic utility of the ISS according to treatment received, ISS was significant in TACE (*p* = 0.0003) and LR (*p* = 0.008). ISS retained its prognostic ability following PS adjustment. In the PS-adjusted model, among patients undergoing LR, ISS of 4 and 5 implied poor prognosis compared to ISS 1 [hazard ratio (HR) 2.13 (95 % CI 0.64, 7.02)], such that ISS was a prognostic score among patients treated with LR [Likelihood ratio test (LRT) *p* = 0.007]. This comparison between ISS 4 and 5 to ISS 1 was evident for patients treated with TACE [HR 3.59 (95 % CI 2.07, 7.57)], (LRT *p* < 0.001, Table 5). On assessing the prognostic value of ISS on either treatment, there was no evidence of a difference in ISS subgroups between LR and TACE groups (*p* = 0.23).

**Table 5:** Propensity score-adjusted Cox proportional hazards model of ISS on overall survival within TACE and LR population and overall likelihood ratio test (LRT), and interaction test to determine effect of ISS between treatments

	TACE intervention, hazard ratio (95 % CI)		LR intervention median OS in months (95 % CI)^+^		*p* value^a^
ISS 1	–	*p* < 0.001	–	*p* = 0.007	0.226
ISS 2	1.30 (0.79–2.14)		1.66 (1.06–2.59)		
ISS 3	1.97 (1.08–3.58)		2.98 (1.61–5.51)		
ISS 4 + 5	3.95 (2.07–7.57)		2.13 (0.64–7.02)		

^a^Log likelihood ratio test of interaction

## Discussion

This is the first large, multi-center study to validate the prognostic ability of the ISS in patients with BCLC-B stage disease, independent of treatment received. Bolondi and colleagues divided BCLC-B stage disease into sub-classifications based on trial results and expert opinion in an effort to reduce heterogeneity in survival in this otherwise disparate patient group. While their method has been validated in a number of papers, this the largest study incorporating both Eastern and Western populations that adheres to the BCLC-B classification. As such this is the first study to explore the use of LR within the BCLC-B classification, albeit in small numbers. PS has been used to reduce confounders between LR and TACE groups, adding to the robust nature of the results obtained.

A plethora of prognostic scores have recently been introduced aiming to improve treatment selection in patients with BCLC-B stage disease [29–31]. These scores such as the Hepatoma Arterial Embolization Prognostic score (HAP score) and Selection for Transarterial chemoembolization Treatment (STATE) score have derived prognostic variables within a cohort and subsequently validated the scores within an external population [30, 31]. The recently proposed ART and HAP scores have attracted significant attention recently particularly as prognostic markers in patients receiving TACE. The HAP score consists of two measures of tumor burden (AFP and size of largest tumor) and two measures of liver function (albumin and bilirubin) [30]. However, the original study included patients with BCLC-A, B and C disease, as well as concerns regarding the independent prognostic ability of bilirubin, may impact on the overall utility of this score. The ART score while useful in determining retreatment with TACE does not contribute to prognostic sub-classification within BCLC-B. Recently Ogasawara and colleagues derived the CHIP score as a means to delineate survival heterogeneity in BCLC-B stage tumors [32]. However, in their paper when compared to the ISS, their novel score showed no real difference in prognostic ability.

The variables included in the ISS are similar to previously identified scores including markers of liver function such albumin, bilirubin, and tumor burden. The main difference with the ISS is that it incorporates three measures of tumor burden; up-to-7 criteria, size of the largest tumor and number of tumors. We report considerable variation in OS from 15.6 to 51 months in our population suggesting that the variables used by Bolondi et al. are useful in delineating prognosis further within this patient group.

A key strength of this study is that we used patient datasets derived from different academic institutions in both Europe and Asia. While TACE is the recommended treatment for BCLC-B patients according to American and European guidelines, in Asian centers, it is not uncommon to propose surgical management [33, 34]. We have shown that ISS retains its prognostic ability in LR or TACE in BCLC-B stage disease prior to and following PS-adjusted analysis. Resection of liver lesions beyond the Milan criteria in BCLC-B population has been shown to improve OS compared to TACE treatment [35], and though beyond the remit of this study, these results suggest that surgical intervention may be a useful treatment modality in a carefully selected population group, and does warrant further investigation in a larger population group within a prospective study design. ISS appears a useful prognostic tool within each treatment category, and there is no evidence of a difference in the effects of ISS subgroups between treatment groups.

However, the inclusion of ‘Quasi C sub-classification’ (ISS 5) and patients with portal vein thrombosis involves a subgroup recognized to possess a poorer prognosis with variable treatment options [36]. While we have demonstrated the prognostic accuracy of the ISS, we have not validated the treatment allocation aspect of the score as proposed by Bolondi et al., an aspect that has not been corroborated in any study. In this context, reflection is required on the use of liver transplant for patients with BCLC-B disease given the poorer overall prognosis of this patient group compared with BCLC-A in the context of global organ shortages. We suggest, therefore, that the role of the ISS is in prognostication rather than as treatment allocation per se.

This is a significant time for the management of HCC as new therapies emerge on the horizon. Useful prognostic tools that improve patient selection are crucial in order to ensure that safe, appropriate and effective therapies are administered in a timely manner. It is evident from this large multi-centered study that the ISS offers a useful tool for clinicians to stratify treatment options, such as TACE and LR, in the BCLC-B population.
